# The Metabolic-Inflammatory Axis in Hypertrophic Cardiomyopathy

**DOI:** 10.1016/j.jacbts.2026.101619

**Published:** 2026-07-27

**Authors:** Katharina J. Ermer, Vasco Sequeira

**Affiliations:** Department of Translational Science, Comprehensive Heart Failure Center (CHFC), University Hospital Würzburg, Würzburg, Germany

**Keywords:** fibrosis, hypertrophic cardiomyopathy, hypertrophy, inflammation, macrophages, metabolism, pioglitazone

**Figure:**
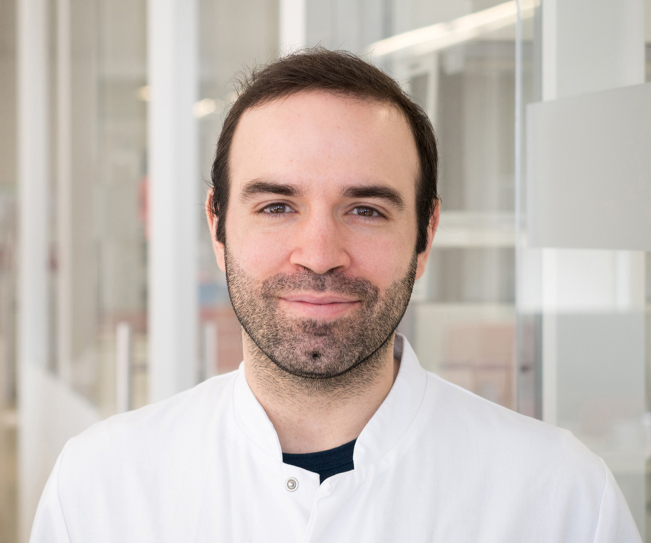

Hypertrophic cardiomyopathy (HCM) is the prototypical sarcomere disease. It affects 0.2% to 0.5% of adults,^1^ with ∼35% carrying a pathogenic sarcomere-gene variant, most often *MYBPC3* or *MYH7*.^2^ These variants destabilize myosin's resting (energy-conserving) state, releasing more heads to form force-producing cross-bridges.^3^ The result is hyperdynamic contraction with high energy demand, slowed relaxation, and a small left ventricular cavity.^1^ When sustained, this energetic cost and downstream oxidative stress promote structural remodeling (hypertrophy, fibrosis).^4^ In obstructive HCM, the high outflow tract gradient raises afterload and adds to this energetic burden.^3^ Current therapies target this mechanical phenotype: β-Blockers, calcium channel blockers, and myosin inhibitors address contractility or its hemodynamic consequences.^1^ Yet nonobstructive HCM lacks disease-targeted therapy, and the ODYSSEY-HCM trial of mavacamten tempered expectations that mechanical unloading alone will halt remodeling.^5^ Another framework is needed, and evidence from other cardiac diseases points toward a metabolic-inflammatory axis.

In this issue of *JACC: Basic to Translational Science*, Pfaller et al^6^ tested pioglitazone (pio), an antidiabetic thiazolidinedione, and its R-form (R-pio) in the α-MHC^719/+^ HCM mouse model, with cyclosporine A to accelerate disease onset. Using echocardiography, histology, RNA sequencing, proteomics, immunohistochemistry, and plasma profiling, they identify 5 pathological layers in untreated α-MHC^719/+^ hearts: 1) left ventricular hypertrophy and interstitial fibrosis; 2) downregulation of transcripts and proteins involved in fatty acid oxidation and mitochondrial respiration; 3) a glucose transporter switch (*Slc2a1* up and *Slc2a4* down, encoding GLUT1 and GLUT4, respectively), implying insulin-independent glucose uptake; 4) altered oxidative stress genes (*Gpx3* up, *Prdx5* down); and 5) local inflammation with macrophage accumulation (IBA1^+^ and CD68^+^ cells) and upregulation of inflammatory and macrophage-recruitment genes (*Ccl2*, *Ccl6*, *Spp1*). Despite the strong myocardial inflammatory phenotype, the plasma cytokine signature was small: Only CCL17 and interleukin-10 increased significantly.

Treatment with pio or R-pio largely normalized these pathological features (Figure 1).^6^ Both agents reduced left ventricular wall thickness and interstitial fibrosis, with R-pio showing a stronger antifibrotic effect (>95% vs ∼65%). At the molecular level, both agents restored mitochondrial pyruvate carrier 1 (MPC1) and the respiratory chain protein profile, attenuated pro-fibrotic and pro-hypertrophic transcriptomic signatures (Wnt, MAPK, SMAD signaling), and reduced myocardial macrophage accumulation. R-pio normalized the circulating inflammatory signature. In wild-type hearts, cyclosporine A alone produces no cardiomyopathy, and neither agent altered wall thickness, fibrosis, or macrophage density, consistent with a disease-specific effect.

**Figure 1:**
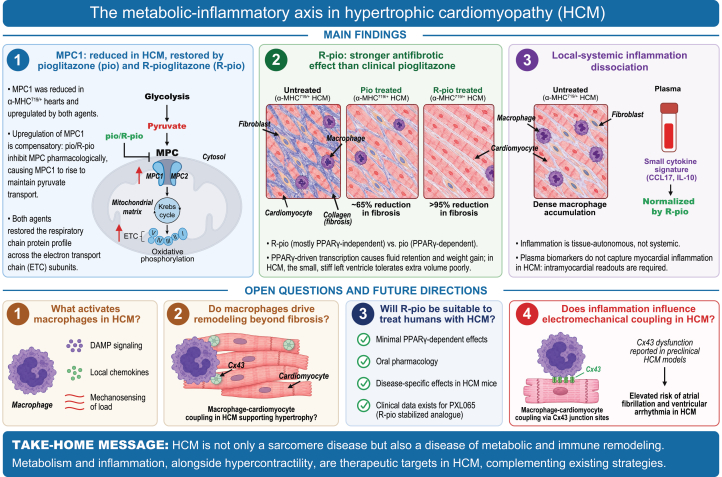
Schematic Overview of the Metabolic-Inflammatory Axis in Hypertrophic Cardiomyopathy (HCM) Cx43 = connexin-43; DAMP = damage-associated molecular pattern; HCM = hypertrophic cardiomyopathy; MPC = mitochondrial pyruvate carrier; pio = pioglitazone; PPARγ = peroxisome proliferator-activated receptor gamma; R-pio = R-form of pioglitazone.

Three findings in Pfaller et al^6^ deserve emphasis.

First, MPC1 emerges as a metabolic therapeutic node. Together with MPC2, it forms the MPC, regulating mitochondrial pyruvate entry. The carrier sits at a metabolic branch point, linking catabolism (glycolysis to oxidative phosphorylation) and anabolism (biosynthetic pathways).^7^ MPC2 was unchanged, whereas MPC1 was reduced in α-MHC^719/+^ hearts and restored by both agents.^6^ Because thiazolidinediones inhibit the MPC complex,^7^ the rise in MPC1 likely reflects a compensatory rather than a direct drug effect: Carrier inhibition prompts MPC1 upregulation to maintain pyruvate transport. In untreated α-MHC^719/+^ hearts, reduced MPC1 protein does not necessarily mean reduced pyruvate flux: Elevated cellular pyruvate, driven by increased glycolysis, can sustain transport through fewer carriers. Multiomic profiling of HCM septal tissue supports this: Pyruvate entry into the Krebs cycle appears preserved despite the shift away from fatty acid oxidation.^4^ In HCM, glucose branches into both glycolysis and anabolic biosynthesis. In sarcomere-positive patients, biosynthetic metabolites (pentose phosphate pathway intermediates and precursors for nucleotide and protein synthesis) increase with septal hypertrophy, consistent with excess glucose being channeled into anabolic pathways that support cardiac growth.^4^ Whether pio/R-pio influences upstream glucose metabolism and this anabolic shift remains untested.

Second, fibrosis in HCM impairs diastolic function and drives arrhythmia risk,^1^ making fibrosis reduction with thiazolidinediones translationally relevant. Thiazolidinediones such as pio classically act via peroxisome proliferator–activated receptor gamma (PPARγ)–driven gene transcription affecting lipid storage and inflammation.^7^ However, because PPARγ activation contributes to fluid retention and weight gain, these adverse effects have constrained thiazolidinedione use in patients with cardiovascular risk.^8^ This is especially a concern in HCM, where a small, stiff left ventricle tolerates extra volume poorly.^1^ R-pio largely bypasses this: It has minimal activating effects on PPARγ yet still shows anti-inflammatory activity^8^ and inhibits the MPC.^7^ These largely PPARγ-independent effects of R-pio are now shown by Pfaller et al^6^ to also be antifibrotic, with a stronger reduction in fibrosis than when using clinical pio (50/50 mixture of R- and S-forms), making it clinically important. PXL065 (stabilized R-pio analogue) is in clinical trials for metabolic dysfunction–associated steatohepatitis,^8^ so a part of the groundwork for a cardiac repurposing trial is in place.

Third, plasma profiling showed a striking dissociation between local and systemic inflammation. Despite dense myocardial macrophage accumulation, the plasma cytokine signature was modest: Only CCL17 and interleukin-10 reached significance, and both were normalized by R-pio.^6^ Inflammation in this model therefore appears tissue-autonomous (driven by cardiomyocyte stress and local chemokine induction, notably *Ccl2* and *Spp1*) rather than systemic. This may explain why plasma biomarkers fail to capture myocardial inflammation in HCM, making intramyocardial readouts a necessity.

Several points from Pfaller et al^6^ warrant consideration. The model uses cyclosporine A (broad immunosuppressant) to accelerate disease onset. Despite this immunosuppression background, macrophage accumulation persists in α-MHC^719/+^ hearts, consistent with a disease-driven inflammatory phenotype rather than a model artifact. Separately, most mechanistic interpretations rest on transcript and protein signatures; functional validation (respirometry for mitochondrial oxidation, stable-isotope tracing of pyruvate flux, fluorodeoxyglucose-positron emission tomography for in vivo glucose uptake) is the next step. Finally, translation from one sarcomeric mouse model leaves open whether the same axis operates across other sarcomere-gene variants or in genotype-negative HCM.

Several open questions stand out (Figure 1).

What activates macrophages in HCM, and do they drive remodeling beyond fibrosis? Pfaller et al^6^ identify myocardial macrophage accumulation and propose that recruited macrophages contribute to interstitial fibrosis, but their upstream triggers and broader remodeling consequences in HCM remain unaddressed. In other cardiac diseases, macrophages promote interstitial fibrosis through fibroblast signaling, physically couple with cardiomyocytes via connexin-43 (Cx43)–dependent contacts, and respond to mechanical stimuli.^9^ In HCM, cardiomyocyte stress is likely the upstream trigger: Metabolic, energetic, and oxidative stress could drive damage-associated molecular pattern signaling and local chemokine induction, whereas hypercontractility may directly activate macrophages. Once activated, macrophages could also support hypertrophic remodeling, as shown in pressure-overload models.^9^ Whether macrophage activation drives structural remodeling in HCM beyond fibrosis and whether pio/R-pio rescue reflects its reversal are questions worth pursuing.

Will the effect translate to humans? R-pio is well suited for translation: minimal PPARγ effects, oral pharmacology, disease-specific effects in HCM mice, and clinical development data for its stabilized analogue PXL065.^8^ A biomarker-guided pilot study in patients with nonobstructive HCM selected by myocardial edema on cardiac magnetic resonance imaging (tissue-level inflammation) and elevated hs-troponin (cardiomyocyte injury), with hs-CRP as a supportive systemic marker, would be a logical next step. The ODYSSEY-HCM trial strengthened the case for adjunctive strategies by demonstrating biomarker-level benefit (N-terminal pro–B-type natriuretic peptide reduction) without significant improvement in exercise capacity^5^: An agent targeting the metabolic-inflammatory axis may be what is missing.

Does inflammation influence electromechanical coupling in HCM? The study by Pfaller et al^6^ was not designed to address whether myocardial inflammatory resolution also affects electrical remodeling. This is nonetheless relevant because HCM carries elevated risk of atrial fibrillation and ventricular arrhythmia.^1^ Cardiac macrophages can influence electrical conduction through Cx43-dependent coupling with cardiomyocytes.^9^ In preclinical HCM models, ventricular arrhythmia susceptibility has been linked to Cx43 dysfunction (reduced and dephosphorylated Cx43), resulting from cardiomyocyte stress.^10^ Whether macrophages directly contribute to such Cx43 remodeling in HCM is unknown, but the convergence of inflammatory and energetic stress in the Pfaller model makes this a clinically relevant future question.

Pfaller et al^6^ add to the evidence that HCM is not only a sarcomere disease but also a disease of metabolic and immune remodeling, with potential pharmacological reversibility across 3 layers of HCM pathogenesis: energetics, inflammation, and structure. The study therefore positions metabolism and inflammation, alongside sarcomere-level hypercontractility, as therapeutic targets in HCM, complementing existing strategies.

## Funding Support and Author Disclosures

Dr Sequeira has received research funding from Bristol Myers Squibb unrelated to this work; and has also received support from the Deutsche Forschungsgemeinschaft (DFG; project nos. 530849567 and 554784412). Dr Ermer has reported that she has no relationships relevant to the contents of this paper to disclose.
